# Fiber Derived Microbial Metabolites Prevent Acute Kidney Injury Through G-Protein Coupled Receptors and HDAC Inhibition

**DOI:** 10.3389/fcell.2021.648639

**Published:** 2021-04-08

**Authors:** Yunzi Liu, Yan J. Li, Yik W. Loh, Julian Singer, Weiping Zhu, Laurence Macia, Charles R. Mackay, Weiming Wang, Steven J. Chadban, Huiling Wu

**Affiliations:** ^1^Department of Nephrology, Ruijin Hospital, Shanghai Jiao Tong University School of Medicine, Shanghai, China; ^2^Kidney Node Laboratory, The Charles Perkins Centre, University of Sydney, Camperdown, NSW, Australia; ^3^Renal Medicine, Royal Prince Alfred Hospital, Sydney, NSW, Australia; ^4^Department of Nephrology, The Fifth Affiliated Hospital of Sun Yat-sen University, Zhuhai, China; ^5^Nutritional Immuno-metabolism Laboratory, The Charles Perkins Centre, University of Sydney, Camperdown, NSW, Australia; ^6^School of Medical Sciences, Faculty of Medicine and Health, University of Sydney, Sydney, NSW, Australia; ^7^Infection and Immunity Program, Biomedicine Discovery Institute, Monash University, Clayton, VIC, Australia

**Keywords:** gut microbiota, acute kidney injury, dietary fiber, prebiotic, SCFA

## Abstract

Short-chain fatty acids (SCFA) derived from gut microbial fermentation of fiber have been shown to exert anti-inflammatory and immune-modulatory properties in acute kidney injury (AKI). However the direct mechanistic link between SCFAs, diet and the gut microbiome is yet to be established. Using the murine model of folic-acid nephropathy (FAN), we examined the effect of dietary fiber on development of AKI (day 2) and subsequent chronic kidney disease (CKD) (day 28). FAN was induced in wild-type and knockout mice lacking G protein–coupled receptors *GPR41*, *GPR43*, or *GPR109A*. Mice were randomized to high-fiber or normal-chow diets, or SCFAs in drinking water. We used 16S rRNA sequencing to assess the gut microbiome and ^1^H-NMR spectroscopy for metabolic profiles. Mice fed high-fiber were partially protected against development of AKI and subsequent CKD, exhibiting better kidney function throughout, less tubular injury at day 2 and less interstitial fibrosis and chronic inflammation at day 28 vs controls. Fiber modified the gut microbiome and alleviated dysbiosis induced by AKI, promoting expansion of SCFA-producing bacteria *Bifidobacterium* and *Prevotella*, which increased fecal and serum SCFA concentrations. SCFA treatment achieved similar protection, but not in the absence of GPR41 or GPR109A. Histone deacetylase activity (HDAC) was inhibited in kidneys of high-fiber fed mice. We conclude that dietary manipulation of the gut microbiome protects against AKI and subsequent CKD, mediated by HDAC inhibition and activation of GPR41 and GPR109A by SCFAs. This study highlights the potential of the gut microbiome as a modifiable target in the prevention of AKI.

## Introduction

Acute kidney injury (AKI) occurs in approximately 10–15% of hospitalized patients (Al-Jaghbeer et al., 2018), with substantial impacts on morbidity, mortality and cost. A variety of pathogenic stimuli in AKI converge on a common cascade of injury-induced events, initiating innate immune responses via damage-associated molecular patterns (DAMPs) which activate Toll like receptors (TLRs) and the NLRP3 inflammasome, forming a vicious cycle of cell death and inflammation driven by pro-inflammatory cytokines, chemokines, and inflammatory cells (Rabb et al., 2016). Characterized initially by an exacerbated innate inflammatory response causing endothelial dysfunction, altered microcirculation, and tubular injury, innate immune cell-derived cytokines facilitate engagement and activation of adaptive immunity. AKI is an independent risk factor for subsequent development of incident chronic kidney disease (CKD), acceleration of pre-existing CKD, cardiovascular disease, heart failure, and death (Coca et al., 2012). Although molecular biomarkers are emerging for risk stratification and early detection, clinically relevant strategies to prevent AKI and associated adverse sequelae are lacking.

The recent surge of interest in gut commensal microbiota and advances in sequencing technologies have greatly changed our understanding of the gut-kidney axis. The potential role of the gut microbiota in the pathogenesis of kidney disorders, through maintenance of intestinal integrity, inflammatory responses, and metabolism has become apparent (Knauf et al., 2019). Short-chain fatty acids (SCFA), gut-derived metabolites produced by bacterial fermentation of non-digestible carbohydrates such as resistant starch, are a key candidate mediator for gut-kidney crosstalk (Sarbini et al., 2014). The most abundant SCFAs generated within the gut are acetate, propionate, and butyrate, of which acetate is present in the highest quantities (mM). In addition to being the main energy source for colonocytes, SCFAs mediate a range of extra-intestinal effects and have the capability to regulate host physiological functions and impact development of immune and inflammatory responses both locally and at distant sites after entering the circulation via active transport mediated by monocarboxylate transporters (Stumpff, 2018). SCFAs exert their effects through binding to metabolite-sensing G-protein-coupled receptors (GPR41, GPR43, and GPR109A) (Tan et al., 2014) or epigenetically via histone deacetylase (HDAC) modulation (Tan et al., 2014).

Experimental models of AKI induced by nephrotoxins (Abdul Hamid et al., 2012; Sun et al., 2013), contrast (Machado et al., 2012), and ischemia (Andrade-Oliveira et al., 2015) have been attenuated by the administration of gut derived SCFAs, highlighting the potential of the gut microbiota as a modifiable target. However, studies to-date have failed to demonstrate a direct mechanistic link between diet, the gut microbiota and response to kidney injury. Drug-induced nephrotoxicity contributes in up to 60% of in-hospital AKI episodes, resulting in substantial morbidity and mortality (Schetz et al., 2005). Murine folic-acid nephropathy (FAN) parallels this common clinical scenario, inducing a toxin-derived AKI through formation of luminal crystals and direct tubular toxicity with subsequent transition to CKD. Here we aimed to obtain deeper insights into how a microbial community responds over time during initiation of AKI and subsequent chronic kidney injury in FAN, and demonstrate how the microbiota’s modifiable capacity can be harnessed through diet to prevent kidney injury.

## Materials and Methods

### Animals

Wild-type (WT) C57BL/6 mice were obtained from the Animal Resource Centre (Perth, WA, Australia). *Gpr41^–/–^, Gpr43^–/–^*, and *Gpr109A^–/–^* mice on a C57BL/6 background were bred and maintained in our facility as previously described (Maslowski et al., 2009). Male mice aged 7–9 weeks were used in all experiments and housed in a specific pathogen free facility within the University of Sydney. All animal care and experiments were conducted in accordance with established guidelines and approved by the Animal Ethics Committees of the University of Sydney.

### Induction of Folic Acid Nephropathy

Folic Acid Nephropathy (FAN) was induced by a single intraperitoneal injection of folic acid (Sigma, FA 8798) at a dose of 200 mg/kg in 0.3M NaHCO_3_ (vehicle). Age and body weight matched controls (non-FAN) received volume and pH matched vehicle.

### Diet and SCFA Treatment

Custom diets were purchased from Specialty Feeds, Australia (Nutritional Parameters: Supplementary Table 1). Mice were fed normal chow (NC) (AIN93G), or a high fiber (HF) modified high-amylose maize starch diet (SF11-025) which is based on AIN93G, commencing 2 weeks prior to FAN induction.

Short-chain fatty acids [150 mM sodium acetate (SA), 150 mM propionate (SP), or 100 mM butyrate (SB)] were dissolved in drinking water pH adjusted to 7.4, and administered to mice fed NC. Control mice received pH-matched control water (Ctrl). SCFA supplemented water was commenced 2 weeks prior to FAN induction and continued *ad libitum* throughout the duration of all experiments.

### Study Design

#### Time-Course Study

To investigate kidney injury events and mechanism, groups of mice were euthanized early (day 2) and late (day 28) after folic acid injection to evaluate acute and chronic kidney injury, respectively.

#### Diet Experiment

To assess the influence of dietary fiber supplementation, we first conducted a pilot study (Supplementary Figure 1). C57BL/6 mice were then randomized to the two different diets commencing 2 weeks prior to FAN induction (Supplementary Table 1) in the following experimental groups with six mice per non-FAN control group at each time point: day 2: (1) NC + FAN *n* = 11; (2) HF + FAN *n* = 7; day 28: (1) NC + FAN *n* = 12; (2) HF + FAN *n* = 12.

#### SCFA Experiment

C57BL/6 mice were maintained on NC and randomized to receive SA, SP, or SB in drinking water commencing 2 weeks prior to FAN induction in the following groups at each time point: day 2: (1) SA + FAN *n* = 10; (2) SP + FAN *n* = 10, (3) SB + FAN *n* = 10, (4) Ctrl water + FAN *n* = 10; with five mice per non-FAN control group; day 28: (1) SA + FAN *n* = 12; (2) SP + FAN *n* = 12, (3) SB + FAN *n* = 12, (4) Ctrl water + FAN *n* = 12; with six mice per non-FAN control group.

#### Mechanistic Experiments

Wild-type, GPR41, GPR43, and GPR109A deficient mice were randomized to diet or SCFA treatment commencing 2 weeks prior to FAN induction in the following groups (*n* = 5–15), evaluated at day 2:

– GPR109A: (1) *Gpr109A^–/–^* + FAN, (2) *Gpr109A^–/–^* + SB + FAN, (3) *Gpr109A^–/–^* + HF + FAN, (4) WT + FAN, (5) WT + SB + FAN, (6) WT + HF + FAN.
– GPR41: (1) *Gpr41^–/–^* + FAN, (2) *Gpr41^–/–^* + HF + FAN, (3) WT + FAN, (4) WT + HF + FAN.
– GPR43: (1) *Gpr43^–/–^* + FAN, (2) *Gpr43^–/–^* + SA + FAN, (3) *Gpr43^–/–^* + HF + FAN, (4) WT + FAN, (5) WT + SA + FAN, (6) WT + HF + FAN.

### Sample Harvest

Blood, kidney tissue, and urine were collected on either day 2 or day 28 after FAN induction. Mouse fecal samples were collected under sterile conditions immediately following extrusion, frozen on dry ice and stored at −80°C.

### Assessment of Kidney Function

Serum creatinine was measured by modified Jaffe method and urea was measured using dedicated Urease/GLDH reagents (Roche) at the Biochemistry Department of Royal Prince Alfred Hospital, Sydney, Australia.

### Histology

Periodic acid-Schiff’s (PAS) and Picro-Sirius Red (PSR) staining were performed on 3 and 5 μm formalin-fixed kidney sections, respectively. Acute tubular injury was assessed on PAS slides in a blinded manner using a scoring system adapted from Martin-Sanchez et al. (2017) containing the following six parameters: loss of brush border, vacuolization, tubular cast formation, tubular epithelial cell edema, cellular infiltration, and tubular dilation. Acute tubular injury was scored on a semi-quantitative zero to three scale for each parameter and the results from each parameter were added to yield the tubular injury score with a maximal value of 18, assessed in at least 15 consecutive fields (×200 magnification). Chronic injury was scored on percentage of renal cortex with tubular atrophy and interstitial fibrosis in 15 randomly chosen, non-overlapping fields at ×200 magnification: 0, none; 0.5, <10%; 1, 10–25%; 2, 26–50%; 3, 51–75%; and 4, >75%. Interstitial collagen deposition was defined as the PSR-positive area, assessed by point counting using a 10 × 10 ocular grid at ×400 magnification in 20 consecutive fields (Li et al., 2020).

### Immunohistochemistry and Quantification

Acetone-fixed frozen sections of kidney and spleen were stained for CD4, CD8, CD68, CD11c, and Ly-6B.2 as described previously (Wu et al., 2020). Endogenous peroxidase activity was blocked with 0.06% hydrogen peroxide, followed by application of a biotin blocker system (DAKO). After blocking with 20% normal horse serum (NHS), primary antibodies: anti-CD4 (BD, 550280), anti-CD8 (BD, 550281), anti-CD11c (BD, 550283), anti-CD68 (ABD Serotec Inc., MCA1957), and anti-Ly-6B.2 (ABD Serotec Inc., MCA771GA) were incubated for 60 min. For visualization of bound primary antibodies, sections were washed then incubated with secondary antibodies: biotinylated anti-rat, anti-hamster, and anti-rabbit IgG (BD Pharmingen). Vector stain ABC kit (Vector Laboratories Inc.) was applied, followed by 3,3diaminobenzidine (DAB) solution (DAKO), and counter-staining with Harris’ hematoxylin.

Immunohistochemistry staining was assessed in a blinded manner by analysis of 20 consecutive high-power fields (×400 magnification) of renal cortex in each section. Macrophage (CD68^+^) and dendritic cell (CD11c^+^) infiltrates were quantified using digital image analysis software (Image Pro Premier 9). T cells (CD4^+^ or CD8^+^) and neutrophils (Ly-6B.2^+^) were counted using an ocular grid and expressed as cells per HPF in a blinded manner as described previously (Wu et al., 2007, 2020).

### Gene Expression Analysis

Total RNA was extracted from kidney tissue with Trizol^®^ (Invitrogen). cDNA was synthesized using oligo d(T)_16_ (Applied Biosystems) primers and the SuperScript III Reverse Transcriptase kit (Invitrogen 18080-044). Taqman Real-Time PCR was employed using the Roche Lightcycler 480 (Roche Applied Science) for the following genes: TLR2, TLR4, NLRP3, ASC, TNFα, IL6, IL18, IL1β, IL4, IL10, IFNγ, CXCL2, CCL2, CXCL10, iNOS, KIM1, MMP2, MMP9, TGFβ1, HDAC1-11, and GAPDH (Applied Biosystems). Results were normalized to GAPDH expression.

### Bacterial DNA Sequencing and Bioinformatics Analysis

Bacterial genomic DNA was extracted from feces, amplified and sequenced at the Ramaciotti Centre for Genomics (University of New South Wales, Sydney, NSW, Australia) as previously described (Li et al., 2020; Wu et al., 2020). Data was deposited in the European Nucleotide Archive (EMBL-EBI) under accession number PRJEB40433. Bioinformatics analysis was performed using the QIIME2 2020.2 pipeline. Paired-end reads were joined using the fast-q algorithm. Demultiplexed sequences were then denoised and ≥97% similar sequences clustered into operational taxonomic units (OTUs) via DADA2. Taxonomies were assigned with BLAST against the Greengenes v13.8 database at 99% sequence identity using q2-feature-classifier algorithm. Taxa present at <0.01% were filtered. Rarefaction analysis was used to compare the adequacy of sequencing depth (Supplementary Figure 2). Data was log2 transformed to account for non-normal distribution of taxonomic count data. Alpha diversity was measured using Shannon diversity. Bacterial community profiles were compared using Weighted Unifrac clustering of OTU abundances. The differential abundance of microbiota species was determined by ANCOM analysis in Calypso and also on complete libraries using DESeq2 model (R package, phyloseq v1.29.0) (McMurdie and Holmes, 2013). Pearson correlation-based network showing relationships between serum creatinine/BUN and bacterial taxa were visualized. Microbial metagenomes were predicted from 16S rRNA gene data using Phylogenetic Investigation of Communities by Reconstruction of Unobserved States (PICRUSt). Identified Kyoto Encyclopedia of Genes and Genomes (KEGG) orthologous groups (KO) with significant differences were visualized using STAMP (Parks et al., 2014).

### SCFA Measurements

Fecal and serum metabolic profiling was performed using ^1^H NMR spectroscopy on a Bruker 600 MHz AVANCE III spectrometer (Bruker BioSpin) and analyzed using Chenomx NMR Suite v8.4 (Chenomx Inc.) as previously described (Li et al., 2020).

### Histone Deacetylase Activity Assay

To determine the activity of HDAC, equal quantities of nuclear fraction proteins (20 μg) were extracted from kidney tissue (Active Motif, 40010), and analyzed using a Fluorometric HDAC Activity Assay Kit (BioVision, k330-100) as described previously (Wu et al., 2020).

### Statistical Analyses

Data are presented as mean ± SD or mean ± SEM. Normally distributed data was analyzed using Student’s two-tailed *t*-tests, or one- or two-way analysis of variance (ANOVA) with *post hoc* multiple comparisons by Tukey’s test. Non-normally distributed data was analyzed using Kruskall–Wallis non-parametric testing with *post hoc* multiple comparisons by Dunn’s test (GraphPad Software). A *P*-value of <0.05 was considered statistically significant.

## Results

### HF Diet Protects Against FA Induced Acute Kidney Injury at Day 2

**FIGURE 1:**
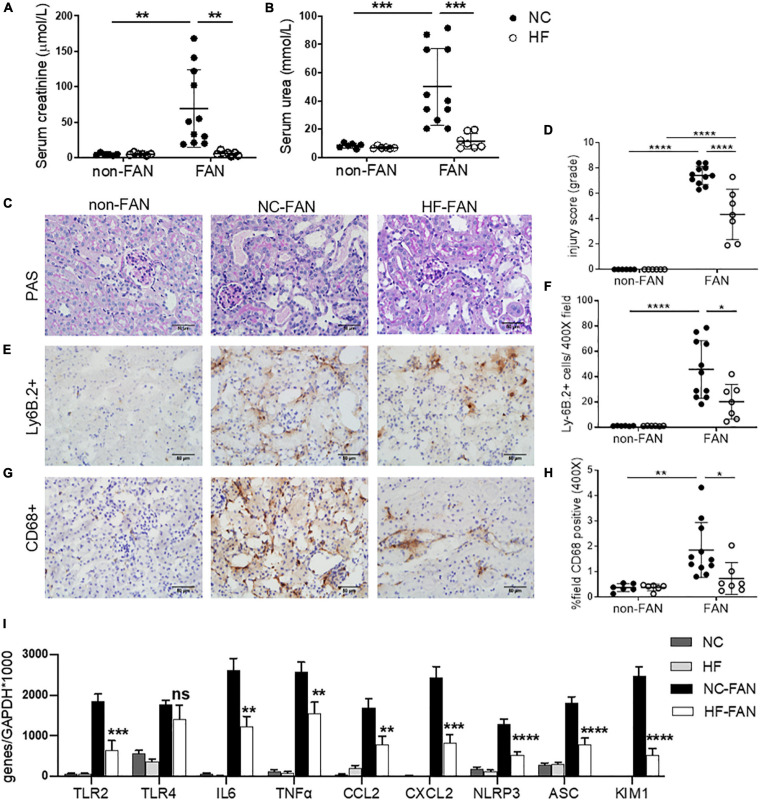
High fiber (HF) diet ameliorates acute kidney injury (AKI) and inflammation in folic acid nephropathy (FAN) at day 2. Folic acid (FA) injection induced AKI in normal chow (NC) fed mice, with peak serum creatinine and blood urea nitrogen (BUN) at day 2. High fiber (HF) fed mice were protected from FA induced AKI with lower serum creatinine **(A)**, BUN **(B)** and tubular injury scores **(D)** compared to NC. **(C)** Representative sections from HF and NC fed FAN and NC fed non-FAN mice at day 2 (PAS stained, ×400 magnification). Representative sections of kidney from mice at day 2 demonstrating increased Ly6B.2^+^ neutrophil **(E)** and CD68^+^ macrophage **(G)** infiltration in NC fed FAN mice compared to controls, which was attenuated by HF feeding **(F,H)**. **(I)** mRNA expression of innate immune receptors (TLR2), pro-inflammatory cytokines (IL6 and TNFα), chemokines (CCL2 and CXCL2), inflammasome components (NLRP3 and ASC), and markers of renal proximal tubular injury (KIM1) were significantly reduced in HF fed mice compared with NC fed FAN controls, as measured by real-time PCR in kidney tissue. NC, vehicle (*n* = 6); HF, vehicle (*n* = 6); NC, FAN (*n* = 11); HF, FAN (*n* = 7). Photomicrographs at 400×, scale bar 50 μm. Data are shown as means ± SD or mean ± SEM; *P < 0.05, **P < 0.01, ***P < 0.001, ****P < 0.0001.

Folic acid induced AKI was characterized by acute renal dysfunction, tubular injury, and tubulo-interstitial inflammation peaking at 48 h after FA injection. To assess the effect of dietary fiber on AKI, we fed WT mice diets containing standard (NC) or enriched amounts of fiber (resistant starch), commencing 2 weeks before induction of FAN. NC fed mice rapidly developed AKI, marked by elevated serum creatinine (SCr 69.3 ± 54.2 vs 5 ± 1.7 μmol/L, *P* < 0.01) and blood urea nitrogen (BUN 50.1 ± 27.2 vs 8.3 ± 1.7 mmol/L, *P* < 0.01) at day 2 post FA injection compared to non-FAN controls (Figures 1A,B). HF fed mice were protected from FA-induced AKI, displaying significantly lower SCr (5.7 ± 3.0 vs 69.3 ± 54.2 μmol/L, *P* < 0.01) and BUN (11.7 ± 5.5 vs 50.1 ± 27.2 mmol/L, *P* < 0.001) compared to those fed NC at day 2, with no difference compared to non-FAN controls (Figures 1A,B). HF feeding also afforded protection as assessed by semi-quantitative histopathological scoring. NC fed mice incurred severe tubulo-interstitial damage, with tubular dilation, endothelial injury, interstitial infiltrate, and intra-tubular cast formation at day 2 post FA injection, which was significantly attenuated in HF fed mice (Figures 1C,D). Non-FAN mice incurred no tubulo-interstitial injury.

### Interstitial Cellular Infiltrates Were Reduced in the Kidneys of HF Fed Mice

Substantial infiltration of Ly6B.2^+^ neutrophils were evident in FAN kidney of NC fed mice at day 2 post FA injection vs non-FAN controls (*P* < 0.001), and this was attenuated in HF fed mice (*P* < 0.05, Figures 1E,F). Similarly, significant CD68^+^ macrophage accumulation was evident in FAN kidney in NC fed mice compared to non-FAN controls, but not in HF fed mice at day 2 post FA injection (*P* < 0.05, Figures 1G,H).

### HF Diet Reduced the Expression of Inflammatory and Tubular Injury Genes Within the Kidney in AKI

Activation of innate immune receptors and the inflammasome by DAMPs, leading to increased expression of downstream proinflammatory genes, is believed to play a major role in the initiation and extension phases of AKI (Leemans et al., 2005; Wu et al., 2007). We next examined the expression of innate immune receptors (TLR2 and TLR4), inflammasome components (NLRP3 and ASC) and downstream inflammatory molecules in the kidney at day 2 post FA-injection by real-time PCR. mRNA expression of TLR2 and TLR4, inflammasome components (NLRP3 and ASC) and downstream pro-inflammatory cytokines (IL6 and TNFα) and chemokines (CCL2 and CXCL2) were significantly upregulated in the kidneys of NC-fed mice at day 2 compared to non-FAN controls. Comparatively, mice fed HF exhibited reduced expression of innate immune receptors and the inflammasome, downstream pro-inflammatory cytokines (IL6 and TNFα) and chemokines (CCL2 and CXCL2) (Figure 1I). This was consistent with markedly reduced CD68^+^ macrophage and Ly6B.2^+^ neutrophil accumulation within the kidney (Figures 1E–H).

KIM1, a specific marker of tubular epithelial cell injury, was profoundly increased at day 2 in the kidneys of FAN mice fed NC, though markedly less so in kidneys of HF-fed mice (Figure 1I), correlating with lower tubular injury scores (Figure 1D).

### HF Fed Mice Were Protected Against Subsequent Chronic Kidney Injury at Day 28

**FIGURE 2:**
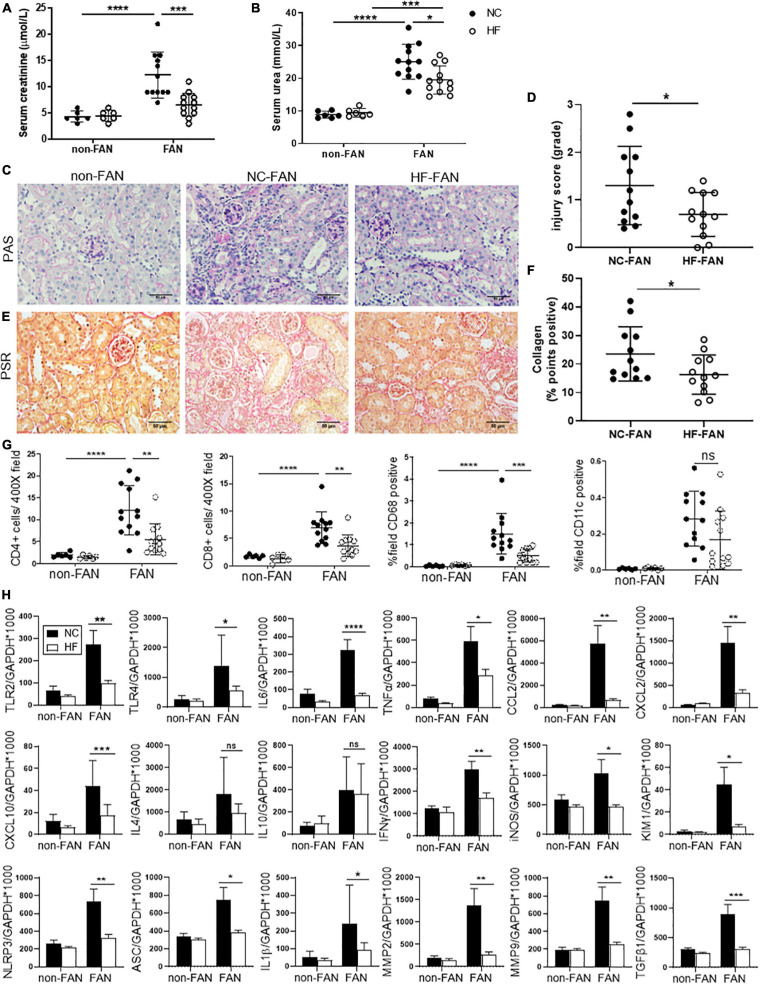
HF fed mice exhibit less chronic inflammation at day 28 after FAN induced AKI. HF fed mice developed less chronic kidney injury at day 28 following FA-induced AKI, with lower serum creatinine **(A)**, BUN **(B)** and histological injury scores assessed on PAS stained kidney sections **(C,D)** compared to NC fed FAN controls. Representative sections of kidney from FAN and non-FAN mice at day 28 demonstrating increased interstitial collagen deposition attenuated by HF feeding on PSR staining **(E,F)**. Quantification of immunostaining for CD4^+^ and CD8^+^ T cells, CD68^+^ macrophages and CD11c^+^ dendritic cells demonstrates significant infiltration in NC fed FAN kidneys. HF feeding significantly diminished T cell and CD68^+^ macrophage accumulation in FAN kidneys, with a trend toward diminished CD11c^+^ infiltrate **(G)**. **(H)** mRNA expression of innate immune receptors (TLR2 and TLR4), pro-inflammatory cytokines (TNFα, IL6, and IFNγ), chemokines (CCL2, CXCL2, and CXCL10), inflammatory mediator (iNOS), inflammasome components (NLRP3, ASC, and IL1β), renal proximal tubular injury marker (KIM1), and fibrosis related genes (TGFβ1, MMP2, and MMP9) were significantly reduced in HF fed FAN mice as compared to NC fed FAN controls. No significant difference was seen in Th2 cytokines (IL4 and IL10) following HF feeding. NC, vehicle (*n* = 6); HF, vehicle (*n* = 6); NC, FAN (*n* = 12); HF, FAN (*n* = 12). Photomicrographs at 400×, scale bar 50 μm. Data are shown as means ± SD or mean ± SEM; *P < 0.05, **P < 0.01, ***P < 0.001, ****P < 0.0001.

The FAN model is characterized by partial recovery from AKI, followed by development of CKD by day 28. NC fed FAN mice exhibited partial recovery in kidney function by day 28, however SCr and BUN remained higher than non-FAN controls (SCr 12.3 ± 4.4 vs 4.3 ± 1.0 μmol/L; BUN 25.1 ± 5.4 vs 8.8 ± 1.1 mmol/L, *P* < 0.001, Figures 2A,B). HF fed FAN mice showed a more complete recovery from kidney dysfunction (SCr: 6.6 ± 2.2 vs 12.3 ± 4.4 μmol/L, *P* < 0.001 and BUN: 19.5 ± 4.3 vs 25.1 ± 5.4 mmol/L, *P* < 0.05, for HF vs NC, respectively), with no difference compared to non-FAN controls (Figures 2A,B).

Normal chow fed FAN mice demonstrated progression to chronic renal injury (Figures 2C,D) and tubulo-interstitial fibrosis following AKI (Figures 2E,F) with persistent renal expression of KIM1 at day 28 (Figure 2H). HF fed mice developed less chronic tubular injury and interstitial fibrosis, evidenced by decreased morphological chronic injury score (Figures 2C,D) and diminished interstitial accumulation of collagen (Figures 2E,F).

The innate immune system activates adaptive immune responses in the extension phase of AKI, promoting chronic inflammation and infiltration of T cells and macrophages, drivers of renal fibrosis (Agarwal et al., 2016). Immunohistochemical assessment revealed significant accumulation of CD4^+^ and CD8^+^ T cells, CD68^+^ macrophages and CD11c^+^ dendritic cells at day 28 in the kidneys of FAN mice fed NC, as compared to non-FAN controls. Accumulation of T cells and macrophages was significantly reduced in kidney from HF fed FAN mice at day 28 (Figure 2G).

### HF Diet Attenuates Chronic Inflammatory Responses Following AKI

Assessment of gene expression relevant to chronic inflammation in NC fed FAN kidney at day 28 revealed significant upregulation of TLR2, TLR4, inflammasome (NLRP3 and ASC), downstream proinflammatory cytokines (IL6, TNFα, and IL1β), chemokines (CCL2, CXCL2, and CXCL10), Th1 cytokine (IFNγ), inflammatory mediators (iNOS) and genes involved in tissue re-modeling (MMP2 and MMP9) and fibrosis (TGFβ1) compared to non-FAN controls (Figure 2H). Less chronic inflammation was observed in the kidneys of FAN mice fed HF compared to NC diet, with persistently lower expression of pro-inflammatory and pro-fibrotic genes (Figure 2H).

### HF Diet Reduces FAN-Induced Dysbiosis and Fosters Expansion of SCFA-Producing Bacteria

**FIGURE 3:**
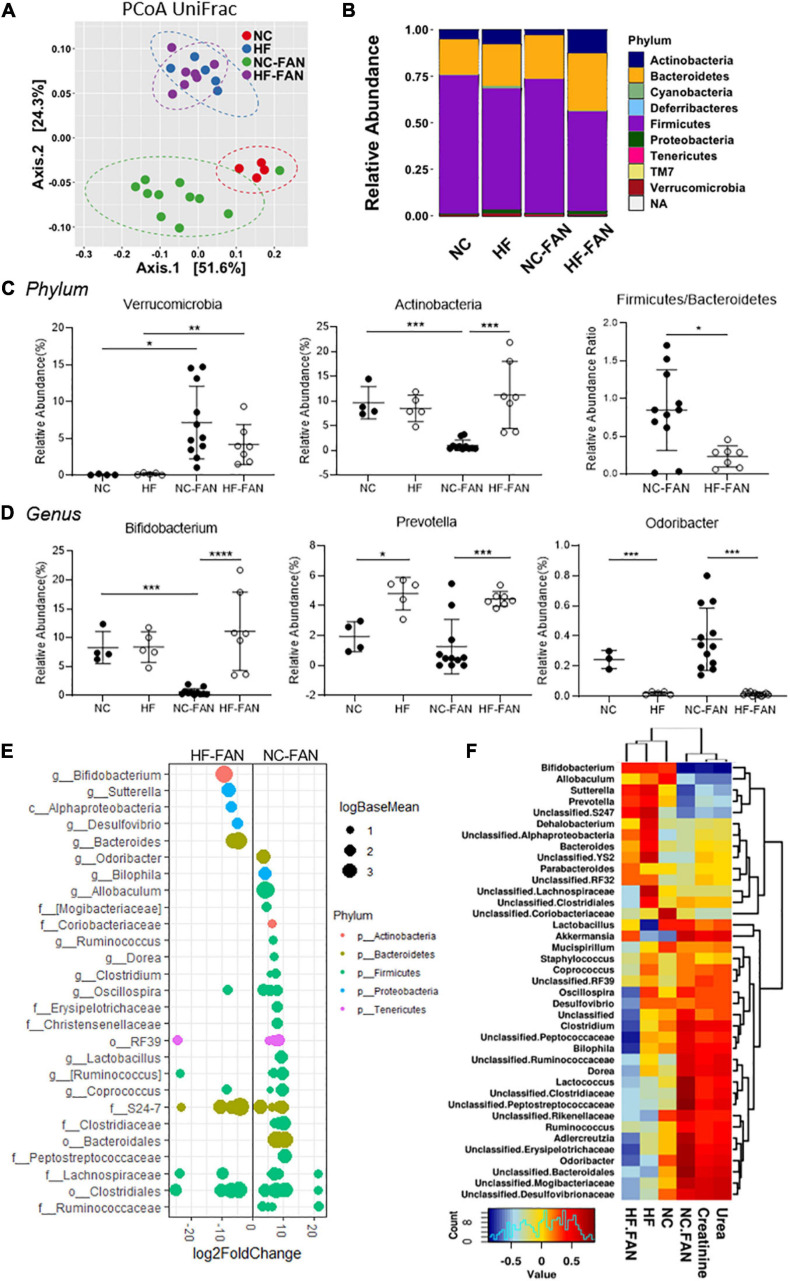
High Fiber diet alters the gut microbial community structure and protects against FAN associated acute dysbiosis at day 2. Fecal DNA analysis using 16S rRNA sequencing was performed on mice fed a NC (FAN: *n* = 11; non-FAN: *n* = 4) and HF (FAN: *n* = 7; non-FAN: *n* = 5) diet at day 2 after FAN induction. **(A)** Principal coordinate analysis of the weighted Unifrac distances demonstrates significant modulation in the microbiota community with cluster separation between diet groups and mice with and without FAN. **(B,C)** Taxonomical composition and relative abundance of different bacteria by ANCOM analysis at the phylum level, depicting “dysbiosis” with expansion of *Verrucomicrobia* at the expense of *Actinobacteria* in NC fed mice, which was modulated by HF feeding. A HF diet suppressed the *Firmicutes* to *Bacteroidetes* ratio. **(D)** At the genus level, NC fed FAN mice had lower levels of the SCFA producers *Bifidobacterium* and *Prevotella*, with relative expansion of the pathobiont *Odoribacter* compared to those fed HF. **(E)** DESeq2 analysis demonstrating differential abundant OTUs (FDR adjusted *P* < 0.01) between dietary groups after FAN induction. OTUs were assigned to their lowest described classification (*y*-axis) and color coded by phylum. Bubble size represents a log fold change in the log base mean of the recorded OTU, with the *x*-axis values demonstrating log2 fold change in relative abundance. **(F)** Pearson-correlation-based heatmap at a genus level, identifying the bacteria associated with increment of serum urea and creatinine. Cool colors represent negative correlation and hot colors represent positive correlations. Data are shown as means ± SD; *P < 0.05, **P < 0.01, ***P < 0.001, ****P < 0.0001.

We next investigated the influence of diet and kidney injury on gut microbiota composition by performing 16S rRNA sequencing of mouse feces at days 2 and 28 following FAN induction. At day 2, there were clear and significant differences in gut bacterial composition associated with FAN, consistent with a disease-related dysbiosis. Principal coordinate analyses of Unifrac Distances showed cluster separation mice with and without FAN (NC vs NC-FAN, *P* < 0.01; HF vs HF-FAN, *P* = 0.032, Figure 3A). This relationship was modified by diet, with additional separation between HF and NC fed groups (NC vs HF *P* < 0.01, NC-FAN vs HF-FAN, *P* < 0.01). Examination of relative abundance at the phylum level found *Bacteroidetes* and *Firmicutes* to be the dominant phyla in all four groups, regardless of diet (Figure 3B). The third most-dominant phylum, however, differed significantly according to diet and disease status. FAN mice fed NC revealed expansion of *Verrucomicrobia* (represented by a single genus, *Akkermansia muciniphila*) at the expense of *Actinobacteria* (Figures 3B,C), a pattern previously reported as dysbiotic (Tan et al., 2016; Wu et al., 2020). In contrast, HF-fed FAN mice exhibited a stable *Verrucomicrobia* population with no difference compared to non-FAN controls (Figures 3B,C). HF diet suppressed the *Firmicutes/Bacteroidetes* ratio and maintained the abundance of *Actinobacteria*, but not *Verrucomicrobia* (Figures 3C,E). At the genus level, expansion of the SCFA producing genera *Bifidobacterium* and *Prevotella*, with relative reduction of pathobionts *Odoribacter, Bilophila, Ruminococcus, Dorea*, and *Clostridium*, was also seen in HF fed mice compared to NC fed controls (Figures 3D,E). Pearson-correlation analysis to identify bacteria associated with severity of renal dysfunction found *Bifidobacterium* to exhibit a strong negative correlation with increment in serum creatinine (*P* < 0.0001, *R* = −0.82) (Figure 3F and Supplementary Table 2). A heatmap based on the Pearson-correlation analysis demonstrated that pathobionts *Odoribacter, Bilophila, Ruminococcus, Dorea, Akkermansia*, and *Clostridium* expanded in NC groups were associated with elevated serum creatinine and BUN, whilst “protective bacteria” expanded in HF fed mice, including SCFA producers *Bifidobacterium* and *Prevotella*, were associated with lower SCr and BUN (Figure 3F and Supplementary Table 2).

We then conducted a gene-centric analysis using the PICRUSt approach to predict functional changes in the gut microbiota that might contribute to improved host renal outcomes. Analysis of 16S-rRNA-inferred metagenomes found a number of metabolic pathways associated with FAN that were modified by diet. Increased activation of pathways associated with damage repair including RNA transcription, repair proteins and alkaloid biosynthesis was seen in FAN mice fed NC compared to HF. Interestingly, we also found greater butyrate and propionate metabolism in FAN mice fed NC compared to a HF diet (Supplementary Figure 3). No difference was seen between diets in non-FAN mice (data not shown), indicating that SCFAs might play a role in reactive anti-inflammatory processes after injury.

At day 28, less disease related dysbiosis was evident, with similar microbial composition between FAN and non-FAN groups as determined by principal coordinate analysis of weighted Unifrac distances (NC vs NC-FAN, *P* = 0.403) and similar alpha diversity. However, significant divergence remained between diet groups (Supplementary Figure 4). At the phylum and genus levels, the alterations in microbial communities induced by AKI at day 2 were relatively maintained at day 28 post FAN induction (Supplementary Figures 4B–D). We used Pearson’s correlation analyses to identify bacteria associated with CKD determined by urea and creatinine increment. HF fed mice maintained a “healthier gut microbiota,” characterized by expansion of the genera *Bifidobacterium* and *Prevotella*, which negatively corrected with creatinine increment (Supplementary Figures 4E,F) and reduction of pathobionts *Odoribacteria* which positively correlated with severity of renal dysfunction and elevated creatinine (Supplementary Figure 4G).

### HF Diet Promotes Sustained Fecal and Systemic SCFA Production

**FIGURE 4:**
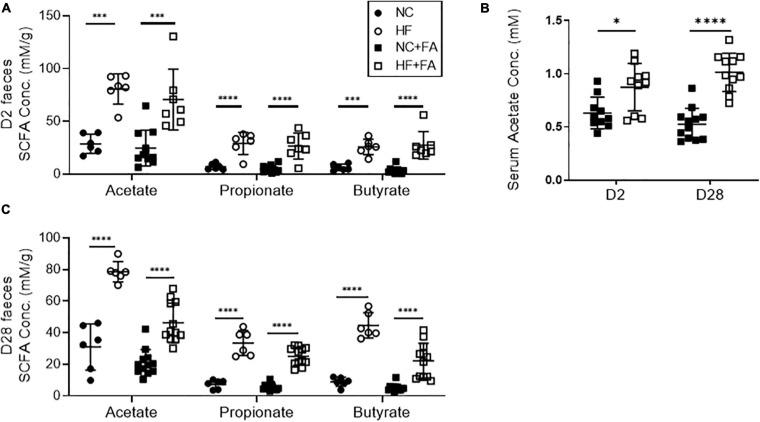
Quantification of SCFA levels in feces and serum by ^1^H-NMR spectroscopy. HF fed mice had significantly higher levels of fecal SCFAs **(A)** and serum acetate **(B)** at day 2 post FAN induction compared to NC fed counterparts. This diet-induced increases in SCFA concentrations were sustained to day 28 **(C)**. FAN (*n* = 7–12); non-FAN (*n* = 6). Data are shown as means ± SD; ^∗^*P* < 0.05, ^∗∗^*P* < 0.01, ^∗∗∗^*P* < 0.001, ^****^P < 0.0001.

As dietary fiber is known to be fermented by the gut microbiota to release SCFAs, we next measured SCFA levels in feces and serum of FAN mice using ^1^H NMR. FAN mice fed a HF diet had significantly elevated serum acetate and fecal concentrations of the three main SCFAs (acetate, propionate, and butyrate), as compared to those fed NC at both day 2 and day 28 (Figure 4).

### SCFA Treatment Protects Against Acute Injury at Day 2 and Chronic Inflammation and Fibrosis at Day 28

**FIGURE 5:**
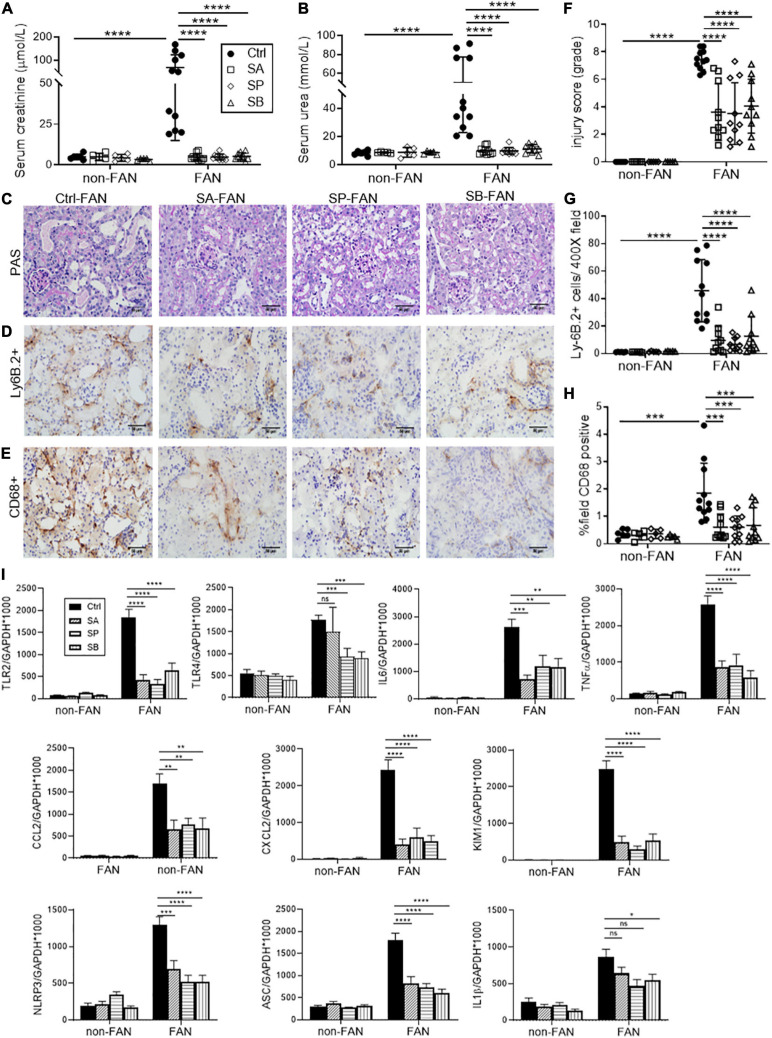
SCFA supplementation attenuates AKI and inflammatory cell infiltrates at day 2. Treatment with SCFAs acetate (SA), butyrate (SB) or propionate (SP) protected against AKI in FAN with lower serum creatinine **(A)**, BUN **(B)** and tubular injury scores **(F)**. Histopathological assessment of PAS stained kidney sections **(C)**, demonstrated protection against kidney damage for all SCFA groups compared to control mice at day 2. Representative sections of immuno-stained kidney for Ly6B.2^+^ neutrophil **(D)** and CD68^+^ macrophages **(E)** demonstrate increased cell infiltrates following FAN induction in control mice, which was significantly diminished by SCFA treatment **(G,H)**. mRNA expression of innate immune receptors (TLR2 and TLR4), pro-inflammatory cytokines (IL6 and TNFα), chemokines (CCL2 and CXCL2), inflammasome components (NLRP3, ASC and IL1β), and the proximal tubule injury marker (KIM1) was diminished by SCFA treatment, as measured by real-time PCR in kidney tissue **(I)**. FAN (*n* = 10); non-FAN (*n* = 5). Photomicrographs at 400×, scale bar 50 μm. Data are shown as means ± SD or mean ± SEM; *P < 0.05, **P < 0.01, ***P < 0.001, ****P < 0.0001.

**FIGURE 6:**
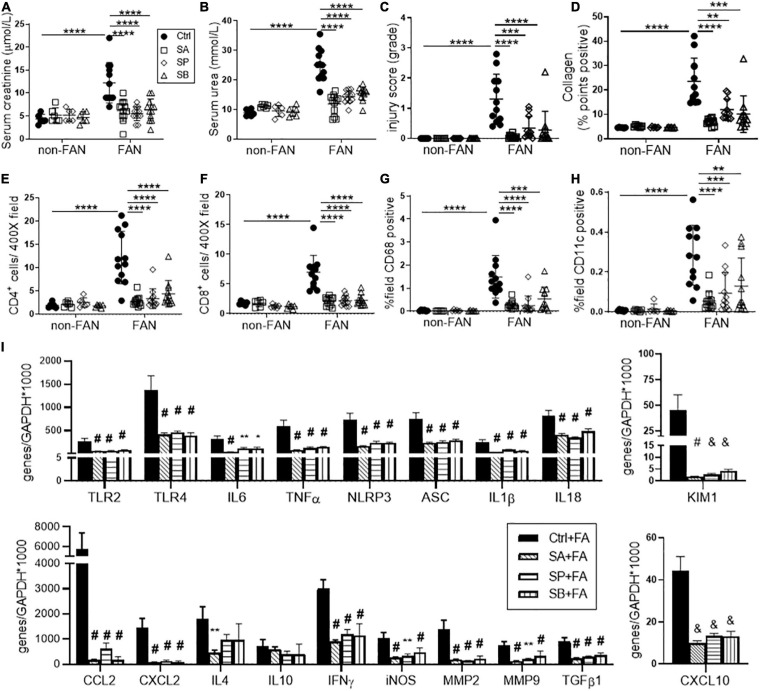
SCFA supplementation resulted in less chronic inflammation at day 28 after FAN induced AKI. SCFA treated mice developed less severe chronic kidney injury, as evidenced by lower serum creatinine **(A)**, BUN **(B)**, histological injury scores **(C)** on PAS staining, and diminished collagen deposition **(D)** on PSR staining at day 28. Reduced CD4^+^, CD8^+^ T cell **(E,F)**, CD68^+^ macrophage **(G)** and CD11^+^ dendritic cell **(H)** infiltration was seen in SCFA treated FAN kidneys, quantified by immunostaining. **(I)** SCFAs diminished mRNA expression of innate immune receptors (TLR2 and TLR4), pro-inflammatory cytokines (IL6, TNFα, and IFNγ), chemokines (CCL2, CXCL2, and CXCL10), inflammasome components (NLRP3, ASC, IL18, and IL1β), inflammatory mediator (iNOS), proximal tubule injury (KIM1), and fibrosis related genes (TGFβ1, MMP2, and MMP9) measured by real-time PCR in kidney tissue at day 28. FAN (*n* = 12); non-FAN (*n* = 6). Data are shown as means ± SD or mean ± SEM; *P < 0.05, **P < 0.01, *** ^&^*P* < 0.001, **** ^#^*P* < 0.0001.

To determine whether changes to SCFA levels could account for the protective effect of HF feeding in FAN, we investigated the effect of SCFA supplementation. Mice were treated with a SCFA [150 mM SA, 150 mM propionate (SP), or 100 mM butyrate (SB)] in drinking water, commenced 2 weeks prior to FAN induction. SCFA treated mice developed less FA-induced acute and chronic kidney injury compared to controls, with all three SCFAs yielding similar degrees of protection against loss of renal function, histological injury and cellular infiltration as HF diet at day 2 (Figures 5A–H) and day 28 (Figure 6). SCFA treatment attenuated both acute and chronic kidney inflammation with downregulated expression of relevant innate immune receptor (TLR2 and TLR4), inflammasome (NLRP3 and ASC), cytokine (IL6 and TNFα), and chemokine (CXCL2 and CCL2) genes in FAN kidneys at day 2 (Figure 5I) and day 28 (Figure 6I). SCFA treated mice showed reduced expression of inflammasome downstream cytokines (IL18 and IL1β), the Th1 cytokine (IFNγ), and iNOS in FAN kidney at day 28 (Figure 6I). Reduced fibrosis, with less interstitial collagen deposition (Figure 6D) and down-regulated expression of TGFβ1, MMP2, and MMP9 was also seen in SCFA treated mice at day 28 (Figure 6I). We have previously demonstrated that SCFA supplementation in drinking water does not alter gut microbiota composition (Tan et al., 2016), supporting a direct effect of SCFA in mediating protection against FAN.

### GPR41 and GPR109A, but Not GPR43 Are Necessary for HF and SCFA Mediated Protection Against FAN

**FIGURE 7:**
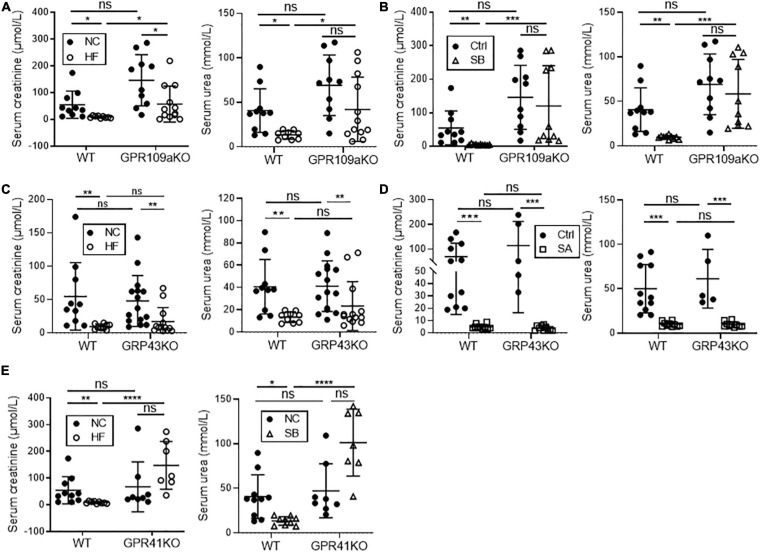
GPR41 and GPR109a play roles in fiber and SCFA mediated reno-protection in FAN at day 2. HF diet provided partial protection against FAN in *Gpr109a^– /–^* mice **(A)**, however butyrate treatment did not improve renal function at day 2 **(B)** with no significant difference in serum creatinine or BUN compared to WT controls. Both HF diet and oral acetate treatment provided full protection against AKI in *Gpr43^– /–^* FAN mice **(C,D)**. HF feeding in *Gpr41^– /–^* FAN mice did not improve renal function at day 2, with no reduction in serum creatinine or BUN compared to WT controls **(E)**. FAN (*n* = 6–12); non-FAN (*n* = 5). Data are shown as means ± SD; *P < 0.05, **P < 0.01, ***P < 0.001, ****P < 0.0001.

To gain mechanistic insights into how SCFAs influence development of FAN, we treated *Gpr41^–/–^, Gpr43^–/–^*, and *Gpr109A^–/–^* mice with a HF diet, or NC diet plus acetate or butyrate supplemented drinking water. Diet or SCFA treatment had no impact on SCr or BUN in non-FAN mice (data not shown). GPR109A, the primary receptor for butyrate, has previously been shown to potentiate anti-inflammatory pathways (Macia et al., 2015). In the absence of GPR109A, butyrate supplementation did not improve renal function in FAN at day 2, whilst HF diet provided partial protection with a small reduction in SCr and BUN compared to WT controls (Figures 7A,B). In contrast, absence of GPR43 yielded no impact on the protective effects of both HF diet and acetate in FAN (Figures 7C,D). As all 3 SCFAs tested are agonists of GPR41, HF diet was chosen to be the intervention in *Gpr41^–/–^* mice. HF feeding did not mitigate renal dysfunction in *Gpr41^–/–^* mice with no reduction in SCr and BUN compared to WT controls at day 2 (Figure 7E). Taken together, these results indicate that the protective effects of a HF diet and the resultant SCFA metabolites were mediated predominantly through GPR41 and GPR109A.

### HF Diet and SCFAs Inhibit Kidney HDAC Activity in FAN

**FIGURE 8:**
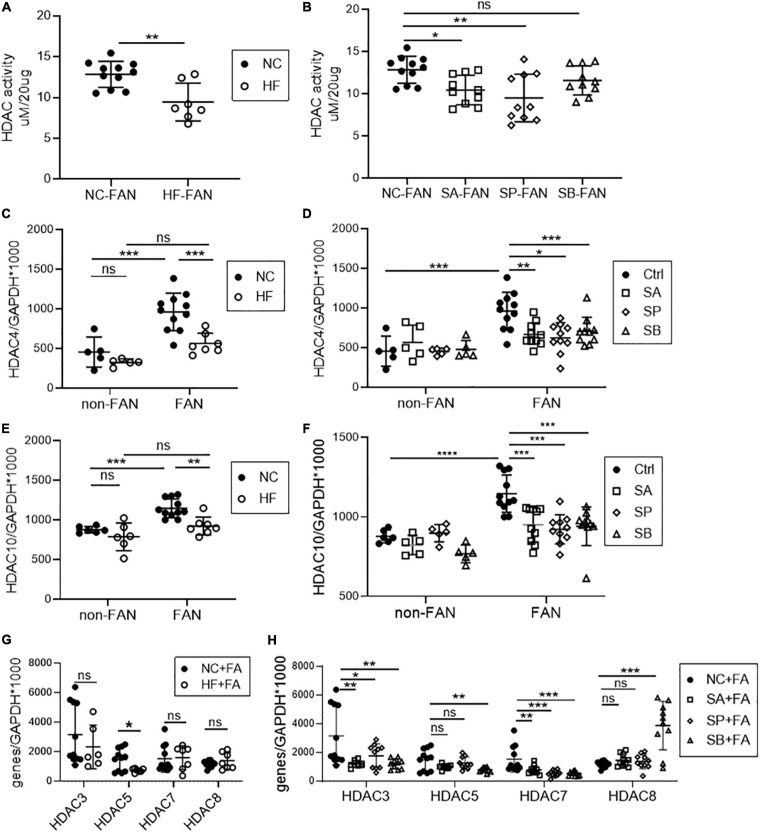
HF and SCFA are involved in epigenetic modification through downregulation of HDAC expression. Downregulation of kidney histone deacetylase (HDAC) activity was seen in HF fed **(A)**, acetate and propionate treated FAN mice **(B)** compared to controls at day 2. **(C–H)** mRNA expression of HDAC subclasses in FAN kidneys. HF diet **(C,E)** and all three SCFAs **(D,F)** reduced kidney mRNA expression of HDAC4 and HDAC10 respectively in FAN. FAN (*n* = 10); non-FAN (*n* = 5). Data are shown as means ± SEM; *P < 0.05, **P < 0.01, ***P < 0.001, ****P < 0.0001.

As natural HDAC inhibitors, SCFAs regulate gene transcription through histone modification. To explore the contribution of HDAC in FAN, we tested global HDAC activity in FAN kidney tissue. Mice fed a HF diet, and those on NC diet treated with acetate or propionate, displayed global inhibition of HDAC activity in kidney tissue at day 2 after FAN induction, however no significant difference was seen with butyrate (Figures 8A,B). To further clarify the differential importance of various HDAC classes, we next examined mRNA expression of 11 subtypes of HDAC in FAN kidney. HF diet and all three SCFAs reduced mRNA expression of HDAC4 and HDAC10 (class IIa and IIb, respectively) (Figures 8C–F). mRNA expression of HDAC3 and HDAC7 was also diminished in the FAN kidney in all three groups of SCFA treated mice compared to controls (Figure 8H). HDAC 5 expression was lower in FAN kidney in HF and SB treated mice while increased expression of HDAC8 was seen in SB treated FAN mice (Figures 8G,H). No reduction was evident in the expression of HDAC 1, 2, 6, 9, and 11 in FAN kidney in response to any treatment. HDAC4 has been shown to be involved in immune regulation, while HDAC10 plays roles in DNA repair, autophagy and anti-cancer responses. These results suggest that dietary fiber may protect against FAN through SCFA mediated HDAC inhibition and downstream changes to gene transcription.

## Discussion

The pathophysiology of AKI shares common pathogenic denominators, including hemodynamic alterations, inflammation, and cell injury, followed by a repair process to restore kidney structure and function (Ronco et al., 2019). The gut microbiota and its metabolites are emerging as therapeutic targets in AKI through their ability to regulate inflammatory and immune responses (Jang et al., 2009; Andrade-Oliveira et al., 2015; Emal et al., 2017). Using the murine model of FAN, we demonstrate a novel bi-directional and modifiable relationship between the gut microbiota, AKI and kidney recovery. AKI alters gut microbial composition, promoting dysbiosis with augmented kidney inflammation and progression to CKD. These changes were ameliorated by a HF diet with protection from AKI and less subsequent renal fibrosis. Gut microbial metabolites (SCFA) were integral to this, exerting effects through binding to GPRs and epigenetic modification as HDAC inhibitors. To our knowledge, this is the first study demonstrating the ability to prevent experimental AKI through dietary modification of the gut microbiota. Overall, these findings provide further mechanistic insights into the gut-kidney axis and highlight its importance across the AKI-CKD continuum.

Regional inflammation within the kidney is the hallmark of this model. Folic acid induced tubular necrosis triggers sterile inflammation, driven by release of DAMPs from injured cells which engage pattern recognition receptors, such as TLRs and NLRs (Rosin and Okusa, 2011), and trigger activation of the inflammasome. Subsequent generation of pro-inflammatory cytokines (including IL1, IL6, IL18, and TNFα) and chemotactic molecules (CXCL2 and CCL2) drives recruitment of neutrophils and macrophages during the early response to injury (Anders and Muruve, 2011; Kurts et al., 2013). The requirement for TLRs and the inflammasome has been demonstrated in studies of mice deficient in TLR2, TLR4, or NLRP3, all of whom were protected against post-ischemic AKI (Leemans et al., 2005; Wu et al., 2007; Iyer et al., 2009). In the acute phase following FAN-induction, we saw upregulation of innate immune receptors and inflammasome components, however expression of TLR2, NLPR3, and ASC were significantly reduced in kidneys of HF fed mice with AKI, identifying these pathways as potentially important in mediating the protective effects of dietary fiber. TLRs and inflammasomes are involved not only in acute responses, but also in chronic inflammation, tissue repair and fibrosis (Artlett, 2013; Anders and Schaefer, 2014; Yiu et al., 2014). The chronic phase of FAN is characterized by a low-grade inflammatory state, consisting of ongoing innate and evolving adaptive immune responses, culminating in fibrosis. T cells and macrophages actively participate in the development of myofibroblasts, which produce extracellular matrix leading to kidney fibrosis (Duffield, 2014). In our study, HF fed mice had less severe AKI, which translated to reduced chronic tubular damage, inflammatory cell infiltration and renal fibrosis. The NLPR3 inflammasome and caspase 1 substrate IL18 have been found to mediate renal fibrosis. We demonstrated sustained suppression of TLR2, TLR4, NLPR3, and ASC mRNA expression in the kidney at day 28, with reduced IL18 and IL1β expression in HF fed FAN mice compared to NC.

Gut microbiota–host immune maladaptation has been implicated in the rising incidence of inflammatory diseases, with recent evidence highlighting the importance of symbiosis and kidney-microbiota crosstalk in health (Knauf et al., 2019). The gut microbiome has been shown to influence susceptibility to AKI, with transfer of “dysbiotic” microbiota low in SCFAs leading to aggravated post-ischemic kidney injury, whilst microbiota depletion with antibiotics provided partial reno-protection (Yang et al., 2020). Similarly, acute renal dysfunction in our model of FAN induced profound changes in the gut microbiome, characterized by significant accumulation of pathobionts, reduction in the abundance of SCFA producing bacteria, and activated damage repair signaling pathways in the gut microbial metagenome as demonstrated using PICRUSt prediction. Manipulation of the microbiome through a diet high in fiber commenced 2 weeks prior to FAN induction led to a reduced *Firmicutes* to *Bacteroidetes* ratio and expansion of SCFA-producing genera *Prevotella* and *Bifidobacterium*, which has been associated with a healthy gut microbial community (Turnbaugh et al., 2006). This diet induced change in microbial composition was associated with protection against development of AKI and subsequent CKD, with less dysbiosis and increased SCFA levels seen in HF fed mice at day 2, which was sustained to day 28. However further studies are required to assess whether the therapeutic potential of HF can be harnessed following an episode of AKI, to prevent AKI-CKD transition.

The relative abundance of *Bifidobacterium* was not only fostered by HF diet, but closely correlated with reductions in acute and chronic kidney dysfunction. *Bifidobacteria* have been tested as a probiotic in interventional trials targeting surrogate endpoints, however evidence of any impact on clinically relevant outcomes remains lacking, with limitations identified in consistent administration due to toxin accumulation in CKD, variability in the intestinal microenvironment and survival rates of probiotics (Koppe et al., 2015). In our study, fiber fostered increased liberation of all three SCFAs with subsequent protection against FAN, pointing toward non-selective prebiotics such as fiber as an alternative to bypass these limitations. The expansion of SCFA producing bacteria seen in HF fed mice and resultant reno-protection highlights the capacity of diet to mediate extra-intestinal effects (Cummings et al., 1987; Murase et al., 1995). Using PICRUSt prediction, we found that butyrate and propionate metabolic pathways were more active within the gut microbial metagenome after AKI in NC fed mice compared to HF, indicating that SCFAs may play a role in reactive anti-inflammatory processes.

Whilst HF fed mice were protected from dysbiosis and AKI, SCFA supplementation, which does not alter gut microbiota composition (Tan et al., 2016), provided similar degrees of kidney protection. This is consistent with the anti-inflammatory properties of SCFAs previously reported in contract-induced (Machado et al., 2012), IRI (Andrade-Oliveira et al., 2015), and toxin-induced (Abdul Hamid et al., 2012; Sun et al., 2013) experimental models of AKI. Caution in the use of SCFAs is warranted however, given that one study of acetate supplementation in C57BL/6 mice from 3 weeks of age led to ureteritis and hydronephrosis by inducing effector (Th1 and Th17) and regulatory T cells (Park et al., 2016), raising the possibility of pleiotropic age and dose dependent effects which require further investigation.

Two predominant mechanisms have been proposed for the operation of SCFAs at a molecular level: (i) as an HDAC inhibitor or (ii) as a ligand for GPRs. We tested the therapeutic effect of HF diet and SCFA supplementation in *Gpr41^–/–^*, *Gpr43^–/–^*, and *Gpr109A^–/–^* mice and found that protection was mediated through GPR41 and GPR109A, but not GPR43. Within the kidney, GPR109A is expressed on podocytes, and participates in reno-protective signaling pathways following butyrate treatment in adriamycin nephropathy (Felizardo et al., 2019). GPR41 is found in human colon mucosal enterocytes, enteroendocrine cells, and smooth muscle cells of the small vessels in kidneys (Tazoe et al., 2009). GPR41 has been shown to inhibit TNF-α-induced MCP-1 expression by modulating p38 and JNK signaling pathways in human renal cortical epithelial cells (Kobayashi et al., 2017). Human renal cortical epithelial cells have been found to express both GPR41 and GPR43, with SCFA treatment of these cells *in vitro* lowering TNF-α induced MCP-1 expression (Kobayashi et al., 2017). Whilst we have previously demonstrated GPR43 to be critical in acetate mediated reno-protection and tolerance in murine models of diabetic nephropathy and kidney transplantation respectively (Li et al., 2020; Wu et al., 2020), this was not seen in FAN induced AKI. The role of GPR43 remains less clear in AKI. Acetate was reno-protective through HDAC inhibition and modulation of T-cell function in a model of sepsis induced AKI (Al-Harbi et al., 2018), whilst both GPR43 dependent and independent mechanisms were contributory in IRI (Andrade-Oliveira et al., 2015). We found that both diet and acetate protected against FA-induced AKI independent of GPR43. This may reflect the complex overlapping biochemical, immunologic, and hemodynamic mechanisms involved in both initiation of and recovery from AKI. Additionally, our knockout mice were not tissue specific, thus it remains to be explored whether HF plays a protective role by modulating immune cells or intrinsic kidney cells through GPR109A and GPR41 in AKI.

In our study, HDAC activity was suppressed in kidney tissues of HF fed mice following AKI, with significant downregulation of HDAC4 and HDAC10 expression. Previous studies have established HDAC4 as playing an essential immunomodulatory role by modifying transcription of immune-related transcription factors, including c-Jun (Gordon et al., 2009), NF-κB and Bcl-6 (Hemmer, 1979; Sandhu et al., 2012), and by regulating the development of various immune cells (Ghisletti et al., 2007; Lu et al., 2015). HDAC10 has been reported to be involved in DNA mismatch repair (Radhakrishnan et al., 2015), cell autophagy (Oehme et al., 2013), and cancer progression (Lee et al., 2010; Fan et al., 2015). However, the functions of HDACs in regulating gene expression and inflammation remain conflicted between different cells and tissues (Liao et al., 2019; Dahiya et al., 2020; Wang et al., 2020). Further studies are required to assess how intrinsic renal and immune cells respond to the suppression of HDAC10 and HDAC4. The relationship between HDAC inhibition and GPRs also remains controversial. Whilst Smith et al. (2013) found HDAC inhibition to be partially GPR43-dependent in colon tissue, others have observed HDAC inhibition independent of GPR-signaling (Aoyama et al., 2010). Further studies are needed to assess the independent or synergistic effects of HDAC inhibition and GPR pathway activation in reno-protection and inflammation.

Our study shows that a high-fiber diet, or supplementation with SCFAs, protects against development of AKI and subsequent kidney fibrosis, and may be a simple and safe means to mitigate susceptibility to AKI. We provide mechanistic insights into the protective actions of dietary fiber, with a specific emphasis on the role of gut derived SCFAs in HDAC and GPR mediated pathways which are integral to inform future translational clinical studies.

## Data Availability Statement

The datasets presented in this study can be found in online repositories. The name of the repository and accession number can be found in the article.

## Ethics Statement

The animal study was reviewed and approved by the University of Sydney Animal Ethics Committee.

## Author Contributions

HW and SC conceived and designed the study. YZL, YJL, YWL, JS, and WZ carried out the experiments. YZL and YJL analyzed and interpreted the data and drafted the manuscript. HW, WW, LM, CM, and SC revised the manuscript. All authors approved the final version of the manuscript.

## Conflict of Interest

The authors declare that the research was conducted in the absence of any commercial or financial relationships that could be construed as a potential conflict of interest.
