# Supplementary material for: Fiber Derived Microbial Metabolites Prevent Acute Kidney Injury Through G-Protein Coupled Receptors and HDAC Inhibition
Source: Front Cell Dev Biol. 2021 Apr 8;9:648639. doi: 10.3389/fcell.2021.648639 (PMC8060457; doi:10.3389/fcell.2021.648639)
Supplement: Supplementary file 1 [file Data_Sheet_1.DOCX]

**SUPPLEMENTARY MATERIALS**

| **Supplementary Table of Contents** | |
| --- | --- |
| **Figure S1** | Pilot study results: HF diet protects against AKI at day 2 in FAN |
| **Figure S2** | Multiple sample rarefaction curve based on 16S rRNA sequencing at day 2 and day 28 |
| **Figure S3** | HF diet alters the functional profiles of gut microbial communities by predictive metagenomics |
| **Figure S4** | HF diet alters the gut microbial community structure and fosters expansion of SCFA producing bacteria, which correlates with renal function at day 28 |
| **Table S1** | Nutritional parameters of experimental diets |
| **Table S2** | Pearson’s correlation coefficients between increment in serum creatinine and bacterial genera in HF fed mice at day 2 |

**
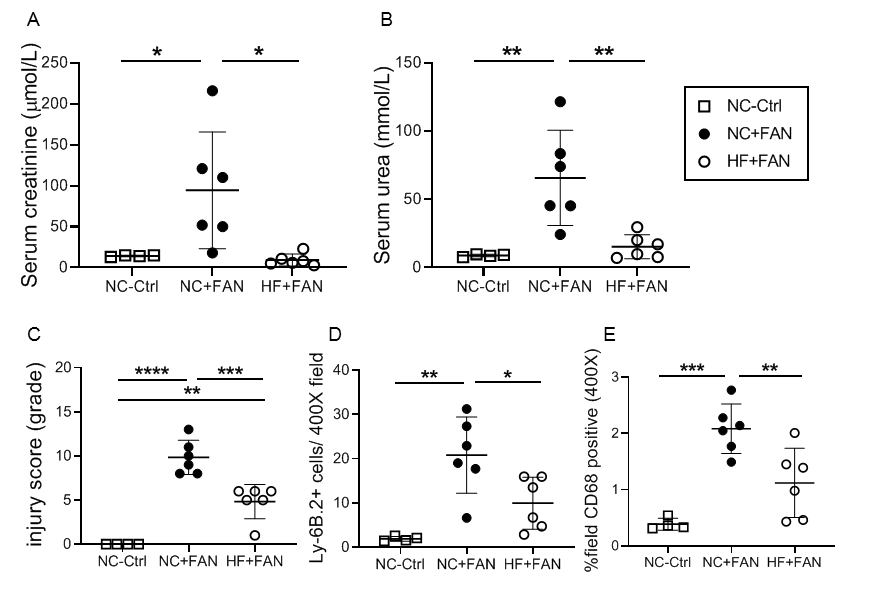
**

**Figure S1. Pilot study results: HF diet protects against AKI at day 2 in FAN.** WT mice were injected with vehicle or FA followed by assessment of renal injury parameters on mice fed NC (FAN: n=6; Ctrl Non-FAN: n=4) and HF (FAN: n=6) at day 2. Mice fed HF were protected from AKI with lower serum creatinine (A), BUN (B), tubular injury scores (C), Ly6B.2^+^ neutrophil (D) and CD68^+^ macrophage (E) infiltration. Data are shown as means ± SD; *P<0.05, ** P<0.01, *** P<0.001, **** P<0.0001.

**
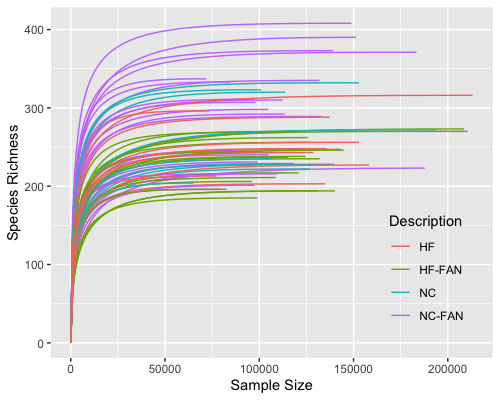
**

**Figure S2. Multiple sample rarefaction curve based on 16S rRNA gene sequencing at day 2 and day 28.** The y-axis shows species richness, and the x-axis shows number of valid sequences per sample. All curves plateau indicates adequate sequencing depth.

**
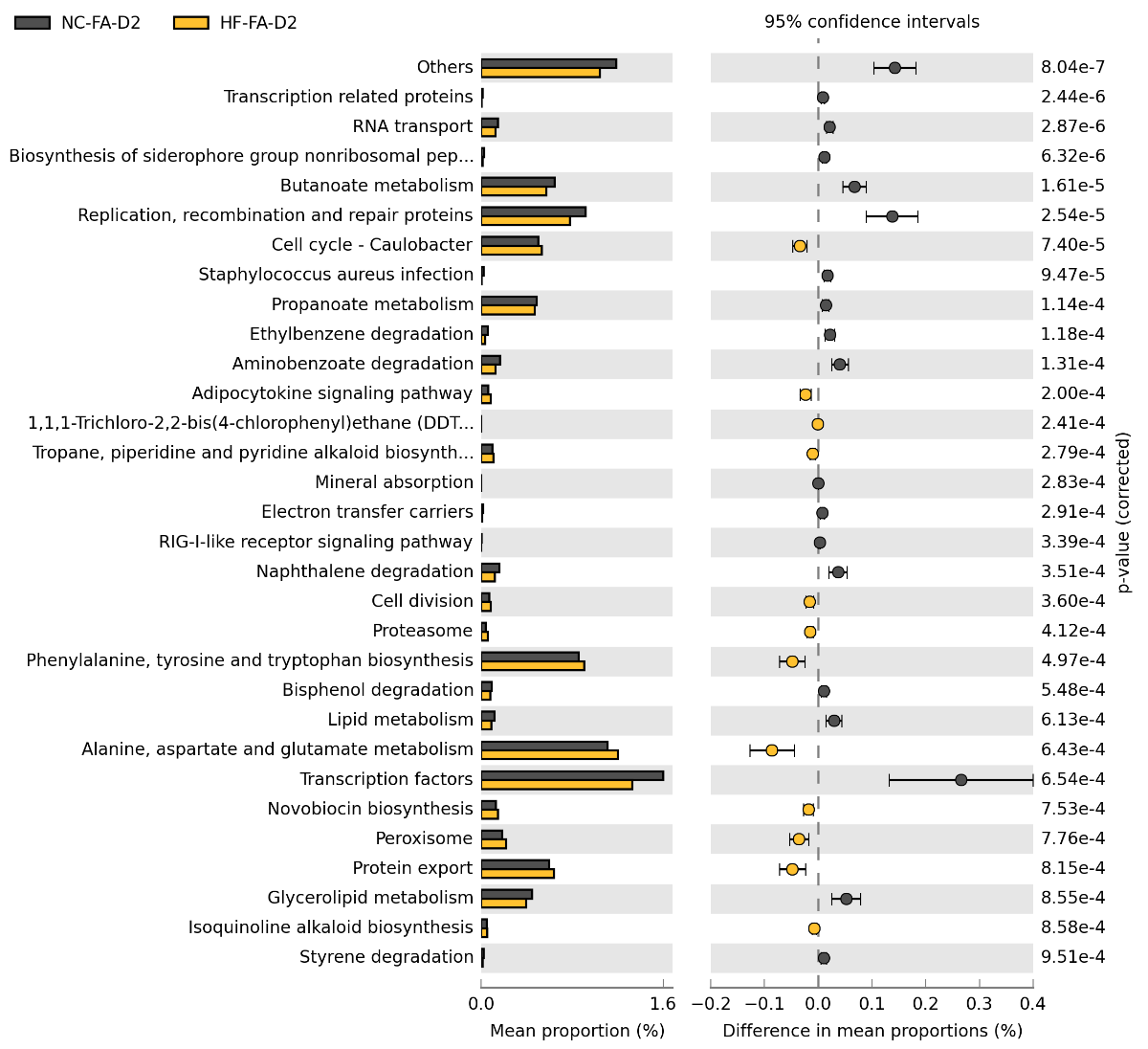
**

**Figure S3. HF diet alters the functional profiles of gut microbial communities by predictive metagenomics.** Differential PICRUSt predicted Kyoto Encyclopedia of Genes and Genomes (KEGG) pathways between diet groups at day 2 after FAN induction, detected by STAMP software. For each comparison, the mean proportion of predicted KEGG pathways (left) and difference in mean proportions (right) were illustrated.

**
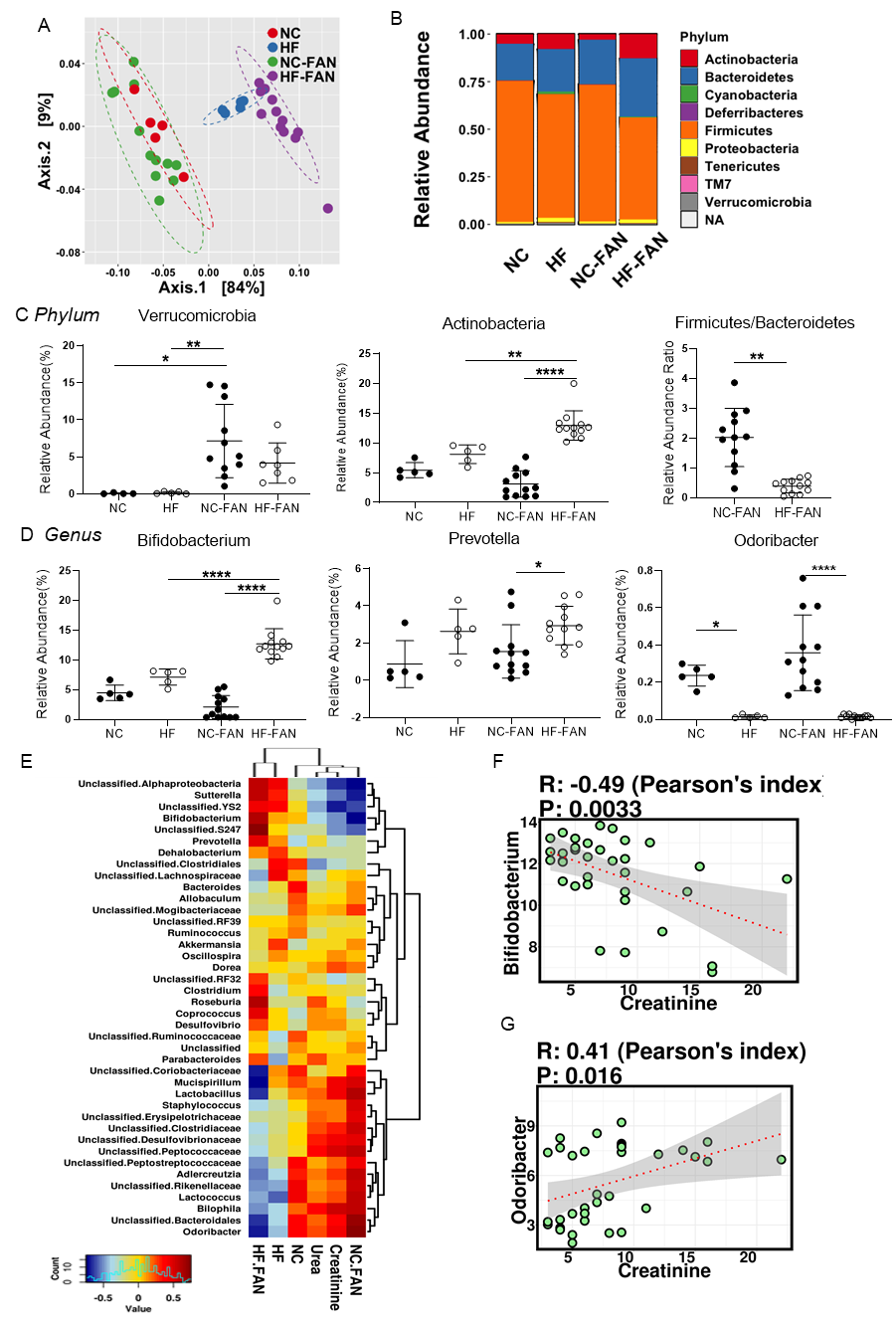
**

**Figure S4. HF diet alters the gut microbial community structure and fosters expansion of SCFA producing bacteria, which correlates with renal function at day 28.** Faecal DNA analysis by 16S rRNA sequencing was performed on mice fed a NC (FAN: n=12; non-FAN: n=5) and HF (FAN: n=12; non-FAN: n=5) diet at day 28 after induction. (A) Principal coordinate analysis of the weighted Unifrac distance demonstrating modulation of the microbiome in mice fed a HF diet after FAN induction, with significant divergence between diet groups after FAN induction (NC vs HF, NC-FAN vs HF-FAN, P<0.01). (B, C) Taxonomical composition and relative abundance of differential bacteria by ANCOM analysis at the phylum level at day 28. (D) At day 28, the HF diet maintains expansion of SCFA producing bacteria of the genera *Bifidobacterium*, *Prevotella* and the reduction in *Orodibacter* seen at day 2*.* (G) Pearson correlation between increment in serum creatinine and differential bacterial genus using ANCOM analysis showing negative correlation of *Bifidobacterium* (R=-0.51, P<0.01) and positive correlation of *Odoribacter*: R= 0.47, P<0.01) with severity of renal dysfunction at day 28. (E) Pearson-correlation-based heatmap at a genus level, identifying the bacteria associated with increment of serum urea and creatinine at day 28. Data are shown as means ± SD; PERMANOVA pairwise testing (A), ANOVA with Tukey post hoc analysis (C, D). *P<0.05, ** P<0.01, *** P<0.001, **** P<0.0001.

| Nutritional Parameter | Control (AING93G) | Resistant Starch  (SF11-025) | |
| --- | --- | --- | --- |
| Crude Fiber | 4.7% | 4.7% | |
| AD Fiber | 4.7% | 4.7% | |
| Starch | 53.6%  (wheat) | 63.6%  (all resistant) | |
| Total Protein | 19.4% | 19.4% | |
| Total Fat | 7% | 7% | |
| Digestible Energy | 16.1MJ/kg | 16.3MJ/kg | |
| Total Digestible Energy From: | | |  |
| Lipids | 16% | 16% | |
| Protein | 21% | 21% | |
| Carbohydrate | 56.8% | 62% | |

^AD, acid detergent

**Table S1. Nutritional parameters of experimental diets.**

| Taxa.(genus) | R | P value |
| --- | --- | --- |
| *Bifidobacterium* | -0.82 | <0.0001 |
| *Prevotella* | -0.38 | 0.048 |
| *Dorea* | 0.56 | 0.002 |
| *Akkermansia* | 0.5 | 0.008 |
| *Odoribacter* | 0.49 | 0.010 |
| *Clostridium* | 0.46 | 0.016 |
| *Bilophila* | 0.42 | 0.031 |
| *Ruminococcus* | 0.36 | 0.068 |

**Table S2. Pearson’s correlation coefficients between increment in serum creatinine and bacterial genera in HF fed mice at day 2.**
